# Impact of individual demographic and social factors on human–wildlife interactions: a comparative study of three macaque species

**DOI:** 10.1038/s41598-020-78881-3

**Published:** 2020-12-15

**Authors:** Krishna N. Balasubramaniam, Pascal R. Marty, Shelby Samartino, Alvaro Sobrino, Taniya Gill, Mohammed Ismail, Rajarshi Saha, Brianne A. Beisner, Stefano S. K. Kaburu, Eliza Bliss-Moreau, Malgorzata E. Arlet, Nadine Ruppert, Ahmad Ismail, Shahrul Anuar Mohd Sah, Lalit Mohan, Sandeep K. Rattan, Ullasa Kodandaramaiah, Brenda McCowan

**Affiliations:** 1 Department of Population Health and Reproduction, School of Veterinary Medicine (SVM), University of California at Davis, Davis, CA 95616 USA; 2 School of Biological Sciences, Universiti Sains Malaysia, 11800 Pulau Pinang, Malaysia; 3 Department of Anthropology, University of Texas at San Antonio, San Antonio, TX 78249 USA; 4 Department of Anthropology, University of Delhi, Delhi, 110007 India; 5 Primate Conservation Group, Oxford Brookes University, Oxford, OX3 0BP UK; 6 Institute of Human Biology and Evolution, Faculty of Biology, Adam Mickiewicz University, 61614 Poznan, Poland; 7 California National Primate Research Center, University of California, Davis, CA 95616 USA; 8 Department of Biomedical Science and Physiology, Faculty of Science and Engineering, University of Wolverhampton, Wolverhampton, WV1 1LY UK; 9 Department of Psychology and the California National Primate Research Center, University of California, Davis, CA 95616 USA; 10 Department of Biology, Faculty of Science, Universiti Putra Malaysia, 43400 UPM Selangor, Malaysia; 11 Himachal Pradesh Forest Department, Shimla, Himachal Pradesh 171002 India; 12 IISER-TVM Centre for Research and Education in Ecology and Evolution (ICREEE), School of Biology, Indian Institute of Science Education and Research Thiruvananthapuram, Maruthamala P.O., Vithura, Thiruvananthapuram, 695551 India

**Keywords:** Ecology, Behavioural ecology, Conservation biology, Urban ecology, Social evolution, Anthropology

## Abstract

Despite increasing conflict at human–wildlife interfaces, there exists little research on how the attributes and behavior of individual wild animals may influence human–wildlife interactions. Adopting a comparative approach, we examined the impact of animals’ life-history and social attributes on interactions between humans and (peri)urban macaques in Asia. For 10 groups of rhesus, long-tailed, and bonnet macaques, we collected social behavior, spatial data, and human–interaction data for 11–20 months on pre-identified individuals. Mixed-model analysis revealed that, across all species, males and spatially peripheral individuals interacted with humans the most, and that high-ranking individuals initiated more interactions with humans than low-rankers. Among bonnet macaques, but not rhesus or long-tailed macaques, individuals who were more well-connected in their grooming network interacted more frequently with humans than less well-connected individuals. From an evolutionary perspective, our results suggest that individuals incurring lower costs related to their life-history (males) and resource-access (high rank; strong social connections within a socially tolerant macaque species), but also higher costs on account of compromising the advantages of being in the core of their group (spatial periphery), are the most likely to take risks by interacting with humans in anthropogenic environments. From a conservation perspective, evaluating individual behavior will better inform efforts to minimize conflict-related costs and zoonotic-risk.

## Introduction

Understanding the factors that influence interactions between humans and wildlife in a shared environment has been a long-standing goal for researchers. The global expansion of human populations and its associated environmental changes mean that wildlife populations are increasingly living in anthropogenically modified landscapes^1–3^. As a result, increasing contact rates and interactions frequently result in conflict, the mitigation of which remains one of the most pressing challenges of the current Anthropocene era^2–5^. To date, most of our knowledge of human–wildlife interactions is limited to visible aspects of such interactions, and to their effects on wildlife systems: human activities such as provisioning animals, habitat fragmentation, and trapping/culling/relocation of animals may affect wildlife population demographics and species decline^3,4,6^, as well as wildlife behaviors related to their foraging and movement, space-use, and social interactions^7–9^. In comparison, we know less about whether, how and to what extent differences in the attributes and behavior of individual wild animals influence their tendencies to engage in interactions with humans and anthropogenic factors^5,10,11^.

Anthropogenic factors present relatively recent, spatiotemporally dynamic environments to wildlife, such that animals have to continuously adjust their strategies and behavior to cope. Thus, the navigation of these environments almost always involve risk-taking behaviors for wildlife, such as entering or moving through anthropogenic landscapes such as agricultural fields and urban settlements, approaching humans, and engaging in antagonistic interactions with humans^2,12,13^. Such behaviors naturally present costs to both wildlife and humans, such as increased risk of injury^1^, negative impacts on wildlife physiology^14^ and human mental health^15^, increased transactional and opportunity costs to humans^15^, and/or vulnerability to cross-species zoonosis (wildlife) and emerging infectious disease (humans)^16,17^. However, such costs may be offset by potential or perceived benefits, such as procurement of high-energy human foods by wild animals^18^ and avoidance of natural predators^19^, and human socioeconomic upliftment through activities like ecotourism^3,15^.

Thus, assessing individual-level variation among wild animals in the context of their interactions with humans is important for multiple reasons. From an evolutionary perspective, assessing inter-individual differences in how animals realize the afore-mentioned costs-benefits tradeoffs, including the frequency and nature of human–wildlife interactions, the behaviors they constitute, and their causal factors, greatly expand our current understanding of animals’ adaptive responses to changing environments^20,21^. From a conservation and public health perspective, understanding whether some individuals (more so than others) are prone to initiate interactions with humans, engage in prolonged interactions, and resort to costly behaviors such as aggression towards humans, will help better inform interventions aimed at conflict mitigation^10^ and/or disease control^22^. From a conflict management perspective, it is especially important to conduct such research in (peri)urban environments, where direct and frequent interactions between wildlife and humans and their associated costs may be especially high (e.g., black bears, *Ursus americanus*^10^; rodents, order Rodentia^23^; moose, *Alces alces*^6^; nonhuman primates, order Primate^24–26^) (reviewed in^27^).

Wildlife behavior in anthropogenic landscapes may be influenced by both intrinsic characteristics of individual wild animals, and extrinsic socioecological factors. With regard to individual wild animals, some intrinsic sociodemographic traits related to an individual’s life-history, specifically age and sex, may influence their tendency to interact with humans^11,13,28,29^. This is because life-history traits are closely inter-linked with energetic demands placed on individuals, which may cause some animals to engage in risk-taking behaviors more than others^11,30^. In most wildlife species, males are more prone to taking risks, as evidenced by their greater exploratory behavior and boldness in personality which are in turn linked to male life-history strategies^30,31^. To increase their competitive ability and thereby their access to females and greater reproductive success, males face high energetic demands in the long-term that are related to retaining characteristics such as larger body sizes and other physical features (e.g., horns, tusks, canines) that afford competitive advantages^32,33^. For these reasons, male animals may be more prone to taking risks to procure high-energy human foods to meet their energetic demands compared to females^18,34^. Indeed, previous studies of wildlife in anthropogenic environments have revealed that males show (1) greater tendencies to forage in agricultural fields (African elephants, *Loxodonta africana*^13^) and urban waste (black bears^10^), (2) have greater access to anthropogenic food (macaques, *Macaca* spp.^34^; Chacma baboons, *Papio ursinus*^35^), and (3) engage in more frequent and antagonistic interactions with people (long-tailed macaques, *Macaca fascicularis*; Formosan macaques, *M. cyclopis*^28,29^). In comparison, females tend to be less prone to engaging with humans, although this might be offset during specific periods of their life-history such as offspring rearing, during which time they may show greater aggression towards humans to protect their offspring, and/or an increased dependence on anthropogenic food to compensate for the high energetic demands of rearing offspring (e.g., rhesus macaques (*M. mulatta*) and captive barbary macaques (*M. sylvanus*):^36,37^).

In group-living animals, aspects of an individuals’ social environment may also influence, or be influenced by, their behavior in anthropogenic landscapes. Studies in a variety of taxa have revealed how anthropogenic factors may influence animal social behavior, by, for example, increasing rates of within-group aggression (e.g., cichlid fish, *Neolamprologus pulcher*^38^; Cuban rock iguanas, *Cyclura nubila*^39^; bonnet macaques, *M. radiata*^40^) and stress-coping affiliative interactions (e.g., Barbary macaques^41^), and decreasing social grooming (e.g., rhesus macaques^42^; long-tailed macaques^43^; bonnet macaques^44^), and connectedness of their social networks (e.g., spotted hyenas, *Crocuta crocuta*^45^; bottlenose dolphins, *Tursiops aduncus*^46^; moor macaques, *M. maura*^11^). In comparison, little research has investigated the inverse effect, namely how the social characteristics of individual wild animals influence human–wildlife interactions^11^.

In group-living animals such as nonhuman primates, individuals’ social characteristics like their dominance rank and connectedness in their social network may influence the way they navigate their environment^47–49^, their access to resources^34,50–52^, and their tendencies to take risks^13,21^. Stemming from this, a handful of studies on nonhuman primates impacted by anthropogenic factors have revealed that individuals’ social characteristics may influence their behavior towards humans. These have revealed somewhat contrasting findings. For instance, high-ranking individuals have been shown to spend more time in human-provisioned areas (e.g. Japanese macaques, *M. fuscata*^53^), and have more access to human foods (urban macaques:^34^), but have also been shown to be less prone to engaging in risky physical interactions with humans (Barbary macaques:^22^), than low-ranking individuals. The impact of social network connectedness is even less clear. In tourist-provisioned Barbary macaques, eigenvector centrality (i.e. the number and strength of both their direct and secondary connections to other group members:^54,55^) was unrelated to macaque presence near tourists, and yet positively correlated with rates of macaque-tourist interactions^22^. In wild but provisioned moor macaques, on the other hand, eigenvector centrality was negatively (rather than positively) correlated to animals’ presence near roads^11^. Such contrasting findings suggest that there may be location- and/or species-dependent differences in whether high-ranking and/or well-connected individuals are more prone to taking risks to engage in interactions with humans^13^, versus engage less on account of the increased time- or energetic demands of maintaining their rank and/or social ties^56^. Moreover, such effects may also be influenced by an individual's spatial position within its group, given that peripheral animals looking to migrate may be more exploratory and therefore, more prone to overlapping with humans and anthropogenic environments^13^. Despite this, spatial position has to date been ignored in studies evaluating the effect(s) of animals’ connectedness or centrality within social networks on human–wildlife interactions^11,22^.

Comparative assessments across different locations and/or wildlife species may generate a better understanding of inter-individual variation in human–wildlife interactions. Indeed, the lack of comparative studies is a major gap in human–wildlife interface research in general^2,3,5^. While potentially providing a more holistic understanding of wildlife behavior from evolutionary and conflict-mitigation perspectives, conducting comparative research is nonetheless challenging given the spatiotemporally variant nature of wildlife (and not to mention human) attributes and behaviors^3,5,12,15^. In other words, such research would require a careful consideration of those aspects of wildlife (and humans) that may be similar, versus different, across locations^3,5^.

Here we implement a comparative approach to examine the impact of the demographic attributes and social characteristics of individual wild animals, on interactions between humans and wild macaques living in (peri)urban environments in India and Malaysia. To this end, we examine data collected by our research team on ten macaque groups representing three species—rhesus macaques in Northern India, bonnet macaques in Southern India, and long-tailed macaques in Malaysia. All three species generally tend to live in multimale–multifemale social groups (but see^57^ for an exception), and yet show marked inter- and intra-specific variation in aspects of their social behavior and social structure^58–60^. Rhesus and long-tailed macaques (both, *Fascicularis* lineage) are more closely related to each other than either is to bonnet macaques (*Sinica* lineage)^59,61^, and have occupied wider geographic ranges and a more diverse range of anthropogenic landscapes than bonnet macaques^26,62–64^. Such similarities and differences in their ecologies and evolutionary histories make these three species well-suited model systems for conducting cross-species comparative assessments of human–wildlife interactions.

For our ten groups of macaques, we examined the impact of inter-individual differences in macaques’ sex, dominance rank, spatial position, and social network eigenvector centrality, on multiple aspects of human–macaque interactions. From a demographic or life-history perspective, we predicted that male macaques, compared to females, engage in more frequent and behaviorally diverse interactions with humans, engage in interactions that involve more exchanges of behaviors within events, show greater frequencies of aggression towards humans, and initiate more interactions with humans, than females. From a social perspective, we expected that dominance rank would be positively correlated to the above aspects of human–macaque interactions, since high-ranking individuals would also be more prone to accessing and monopolizing anthropogenic food resources^34^. Given our previous findings that social grooming among these urban macaques was subject to time-constraints imposed by their interactions with humans^42–44^, we expected that spatial position and social network connectedness would negatively impact the aforementioned aspects of human–macaque interactions. In particular, eigenvector centrality, through its evaluation of individuals’ direct and secondary network connectedness, is an apt measure to determine whether social connectedness leads to a decrease in aspects of human–macaque interactions on account of animals’ spending more time socializing (as we predict here), versus to an increase in such interactions on account of individuals with greater social connectedness or ties of support being more prone to taking risks (alternative hypothesis). Thus, we predicted that spatially peripheral individuals, and/or individuals that possessed the least number and strength of social connections within their grooming network (eigenvector centrality), will engage in more frequent, behaviorally diverse interactions, engage in greater behavioral exchanges within events, greater frequencies of aggression towards humans, and initiate more interactions with humans, compared to more central individuals. Adopting a comparative approach, we also examined whether, and the extent to which, the above predicted patterns were similar versus different across three macaque species.

## Methods

### Study site and subjects

We observed adult individuals in each of ten groups of urban/peri-urban macaques ranging from temperate areas in Northern India, to tropical environments in Southern India and Malaysia. In the Northern Indian city of Shimla (31.05 N, 77.1 E), we observed four groups of rhesus macaques from July 2016 to February 2018, three groups at a temple and surrounding forested area and one group near the city mall area (for more details on the study site see^42,65^). In Malaysia, we observed four groups of long-tailed macaques in Kuala Lumpur (3.3 N, 101 E), from September 2016 until February 2018. Two were observed at a large Hindu temple frequented by tourists, and the other two in a recreational park on the outskirts of the city (for more details on the study site see^34^). From July 2017 until May 2018, we studied two groups of bonnet macaques in the Thenmala Dam and Ecotourism Recreational Area (8.90 N, 77.10 E) located at the outskirts of the small town of Thenmala within the state of Kerala in Southern India (for more details on this study site see^44^). More details related to the group sizes and compositions for all ten groups may be found in^34^.

### Data collection

Data collection followed a standardized protocol that was implemented across all field sites (see^65^ for details). Specifically, we collected data on pre-identified adult individuals of each macaque group for 2–3 days per week, using 10 min focal animal sampling sessions^66^. These sessions were conducted between 0900 and 1700 h. Individual macaques were observed in a predetermined, randomized sequence. In each focal session, we recorded all human–macaque interactions, and macaque–macaque agonistic and affiliative grooming interactions in a continuous manner. We defined a human–macaque interaction as any behavior initiated by the focal animal towards humans, or by a human towards a focal animal with a subsequent reaction from the initial recipient (for a complete ethogram of macaque and human behaviors, see^65^). An agonistic interaction was defined as dyadic aggression which elicited a submissive response from the recipient, or a dyadic submissive displacement event in a non-aggressive context (for a full list and definitions of macaque–macaque aggressive and submissive behaviors, see^65^). For social grooming, i.e. the cleaning or manipulation of the fur of another individual, we recorded both the instance as well as the start and end times of each grooming bout to record its duration (details in^65^).

**Figure 1:**
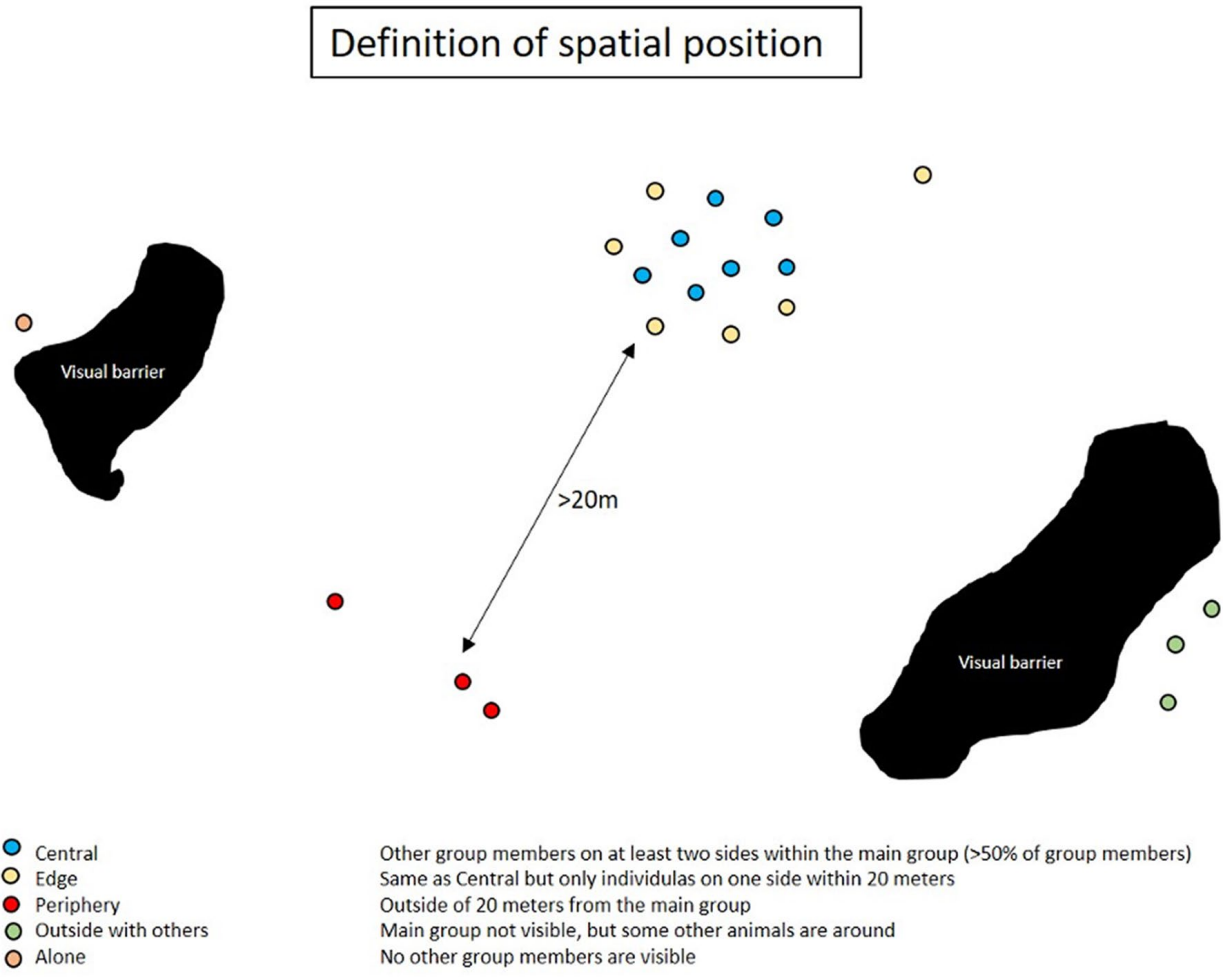
Definitions and estimation of macaques’ spatial position with respect to their group. Figure was created using Microsoft PowerPoint (Version 16.42).

In addition to collecting continuous data on interactive behaviors, we conducted instantaneous sampling once every two minutes within our 10-min focal session resulting in five samples per session. During these, we recorded the focal animal’s spatial position relative to the center of the group. Specifically, we recorded the individual’s position as ‘central’ when other individuals were around it on at least two sides within 20 m. If an individual had the majority of the group (> 50% of the individuals) only on one side but within no more than 20 m, we recorded its position as ‘edge’. If the majority of the group was visible but at least 20 m away from the individual, its position was recorded as ‘peripheral’. If the majority of the group was not visible but few other group conspecifics were around the individual, its position was recorded as ‘outside with others’. If no other group conspecifics were visible around the individual, we recorded its position as ‘alone’ (see Fig. 1).

All data were entered directly into Samsung Galaxy Tablets using customized data forms created in HanDBase^®^ application (DDH software). From these they were exported and tabulated into MS Excel and MS Access databases on a daily basis. All observers within and across sites passed inter-observer reliably tests using Cohen’s kappa, and were deemed reliable when Cohen’s kappa was > 0.85^67^.

### Computation of human–macaque interactions and social factors

Data from individuals with less than 300 min of observation time were excluded from the analyses. The choice of 300 min was based on a clear break the distribution of total observation times across individuals, which showed that the majority of animals (319 out of 342, or 93%) were observed for 300 or more minutes. A minority of individuals which either died or dispersed from the study group(s) soon after the commencement of observations on them (23 out of 342, or 7%) were removed from the analyses. Thus, we analyzed data from 319 individuals distributed across the ten macaque groups^34^. Details of group compositions, sizes, and sex ratios for all ten groups are provided in^34^.

For each focal individual macaque, we calculated various aspects of their interactions with humans. Specifically, we calculated (i) *frequency of human–macaque (HM) interactions*^42–44^, and (ii) the *diversity of HM interactions* defined as the number of unique behaviors shown by the focal towards humans relative to the total number of unique behaviors by all focal animals across the entire observation^65^. Thus, a focal animal that primarily showed aggression towards humans would have a lower diversity score than an animal in its same group that avoided humans, engaged in begging humans for food, and showed aggression towards humans. We also calculated (iii) the *complexity of human–macaque interactions* as the average number of individual behaviors exchanged by the two participants (macaque and human) across all their interaction events. The simplest human–macaque interactions only consisted of two behaviors, an action and a corresponding reaction, for example an approach from the initiator and a subsequent avoid from the recipient. The complexity would therefore increase when humans or macaques engage in more interactive behaviors within an event (i.e. a continuous exchange of interactions between two participants). We further calculated (iv) *macaque-to-human aggression* as the frequency of aggression given by the focal towards humans, and (v) *macaque initiation of human–macaque interactions* as the proportion of their total HM interaction events that were initiated by focal animals.

For each macaque, we also computed its social characteristics, specifically its dominance rank, overall spatial position in the group, and connectedness or eigenvector centrality in the social grooming network. First, we determined dominance rank from dyadic dominance interactions with clear winner/loser outcomes using the package ‘Perc’ in R (R Development Core Team^68^)^69^. We calculated individual ranks based on hierarchies reconstructed separately for males and females. We further standardized ordinal ranks to account for group size and created a rank index ranging between zero and one, indicating the top- and bottom-ranking macaque, respectively. For the data on spatial location, we assigned an ordinal scale ranging from 0 (for ‘alone’) to 5 (for ‘central’) and calculated a mean ordinal position for every individual from across all its spatial position values. From the data on dyadic grooming interactions, we reconstructed weighted, undirected social networks for each macaque group. In these, nodes were individual macaques, and edge-weights were proportions of time spent grooming (given plus received) between each pair of individuals. The latter were calculated taking into consideration the observation times of each pair of interactants during the course of their overlapping tenure in the group. From these, we calculated rescaled values of grooming eigenvector centrality, i.e. the number and strength of both their direct and secondary grooming connections^54,55^, using the ‘Statnet’ package in R^70^. All macaque social networks were visualized using Cytoscape v3.7.2.^71^.

### Data analyses

We used Generalized Linear Mixed Models (GLMMs) to test our predictions, with various aspects of human–macaque interactions described above being set as outcome variables. In these, we treated total *number or frequency of HM interactions* (i), the *diversity* of interactions (ii), and *frequency of macaque-to-human aggression* (iv) as count data, and included the total observation time of each individual as an offset variable in the corresponding models. We also treated the number of *HM interactions initiated* by macaques (v) as a count, but included the total number of HM interactions (rather than observation time) as an offset variable in these models in order to gauge effects on the *proportions* of macaque-initiated HM interactions. Models related to the frequency of HM interactions (i), macaque-to-human aggression (iv), and the proportion of macaque-initiated HM interactions (v) were all over-dispersed. Thus, we ran negative binomial models in these cases. On the other hand, diversity (ii) was not over-dispersed, so we used a Poisson link function. Finally, since complexity (iii) of HM interactions (calculated as a mean value for each macaque from across all its HM interactions) followed a normal distribution, we used a Gaussian error distribution for these models.

For each outcome variable, we ran six GLMMs (Supplementary Table S1). In every model, we included ‘Group ID’ as a random factor in order to account for between-group, within-site differences in macaque behavior and overall exposure to human activity. Where relevant, we included observation time or the frequency of HM interactions as offset variables (see previous paragraph). The first was a ‘null’ model which included just the random factor and (if relevant) an offset variable. In a second model we included ‘Species’ as well as our primary predictor variables (i.e. sex, dominance rank, spatial position, and social network centrality) as main-effects. To investigate cross-species differences in the effects of these predictors on our outcome variables, we ran four additional models containing interactions between species and each of our four primary predictor variables.

Within each set of six models, we used AICc scores to choose the ‘best-fit’ model^72,73^. Specifically, we compared models with just main-effects terms with the corresponding ‘null’ model. Since models that included interactions with species were essentially more complex, or less parsimonious versions of the model with just main-effects, we compared their AICc scores to the latter rather than to the ‘null’ models^74^. In the results, we report both the ‘best-fit’ model from each set that had the lowest AICc score^72,73^.

For all best-fit models, we checked various diagnostics of model validity and stability (Cook's distance, DFBetas, DFFits, and Variance Inflation Factors; distribution of residuals, residuals plotted against fitted values). None of these tests indicated obvious influential cases, strong collinearity among our predictor variables, or obvious deviations from the assumptions of normality and homogeneity of residuals^75^. All significance levels were set to two-tailed p values < 0.05. We analyzed the data in R using the ‘lme4’^76^ and ‘glmmADMB’^77^ packages.

### Ethics declaration and approval for animal experiments

The protocols used in the study were approved by the Institutional Animal Care and Use Committee (IACUC) of the University of California, Davis (protocol # 20593). The research was performed strictly in accordance with the guidelines and regulations drafted in this protocol. Observers did not engage in any contact or non-contact interactions with the animals while recording their natural behavior. No biological samples were collected. Since exclusively observational data were collected on both the monkeys and humans, with no identifying information collected on the humans and no interactions between the experimenters and the humans, no human subjects were enrolled to directly participate in this study. This protocol, along with the guidelines and regulations, was designed in consultation with the Himachal Pradesh Forest Department and the Indian Institute of Science Education and Research Thiruvananthapuram in India, and Universiti Putra Malaysia and Universiti Sains Malaysia in Malaysia. They complied with the legal requirements of India and Malaysia.

## Results

For all five aspects of human–macaque interactions, we identified a single best-fit model. This was either the model with just main effects which was significantly better-fit (i.e. ∆AICc < 2) than the corresponding ‘null’ model, or a model that included an interaction term with ‘species’ that was significantly better-fit than the model with just main effects (Supplementary Table S1). For frequency of HM interactions (i) and complexity (iii), the best-fit model was one that included an interaction between species and social network connectedness (Supplementary Table S1). For diversity (ii), macaque-to-human aggression (iv), and % interactions initiated by macaques (v), the best-fit model was the one that included just the main effects (Supplementary Table S1).We summarize the main findings from these best-fit models below:

### Frequency of HM interactions

**Figure 2:**
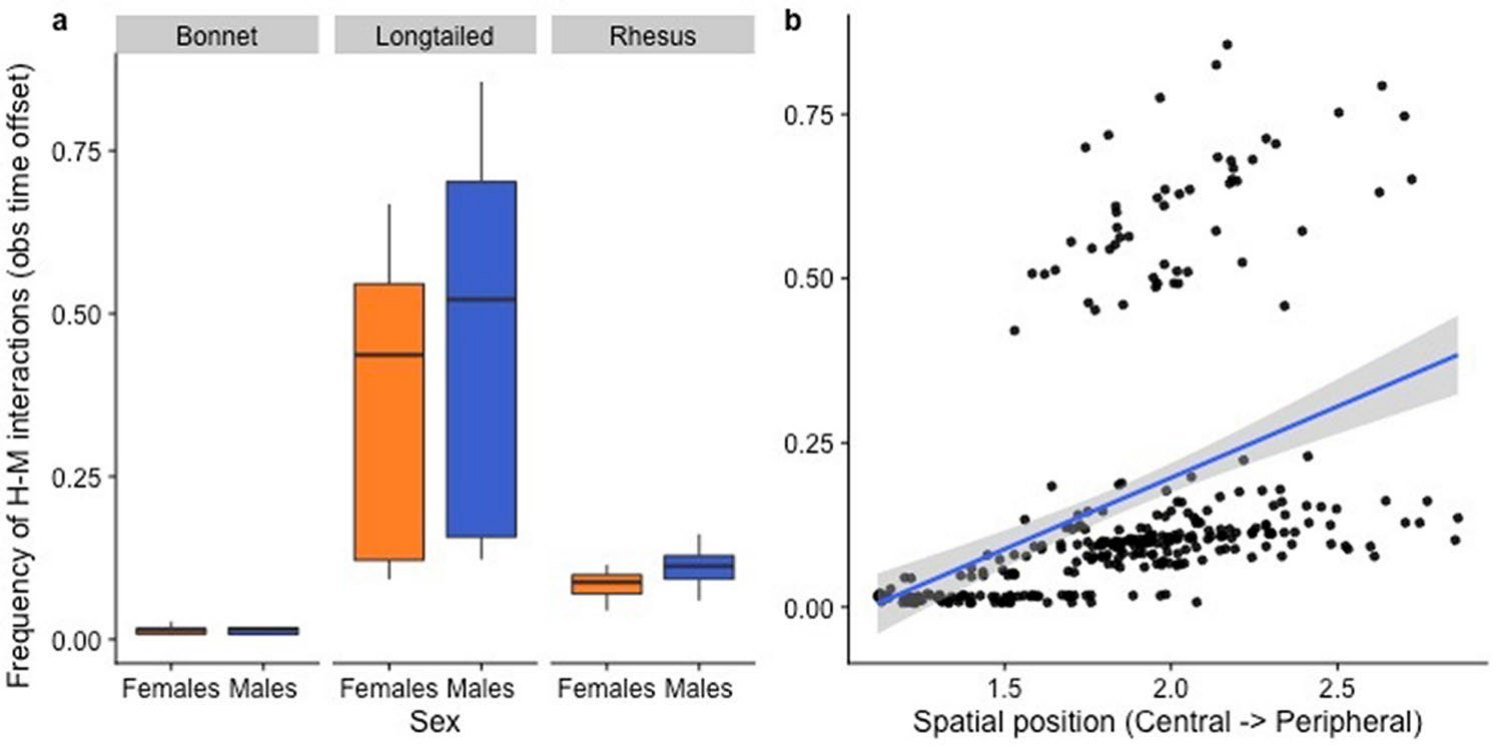
Effect of sex, species, and spatial position on the frequency of human–macaque interactions. The second grouping in the scatterplot represents individuals from two longtailed macaque groups included in the study that were observed at Batu Caves, where they experienced an exceptionally high frequency of human–macaque interactions (Y-axis)^43^. Figure was created using R (Version 3.6.1: https://www.r-project.org/).

**Figure 3:**
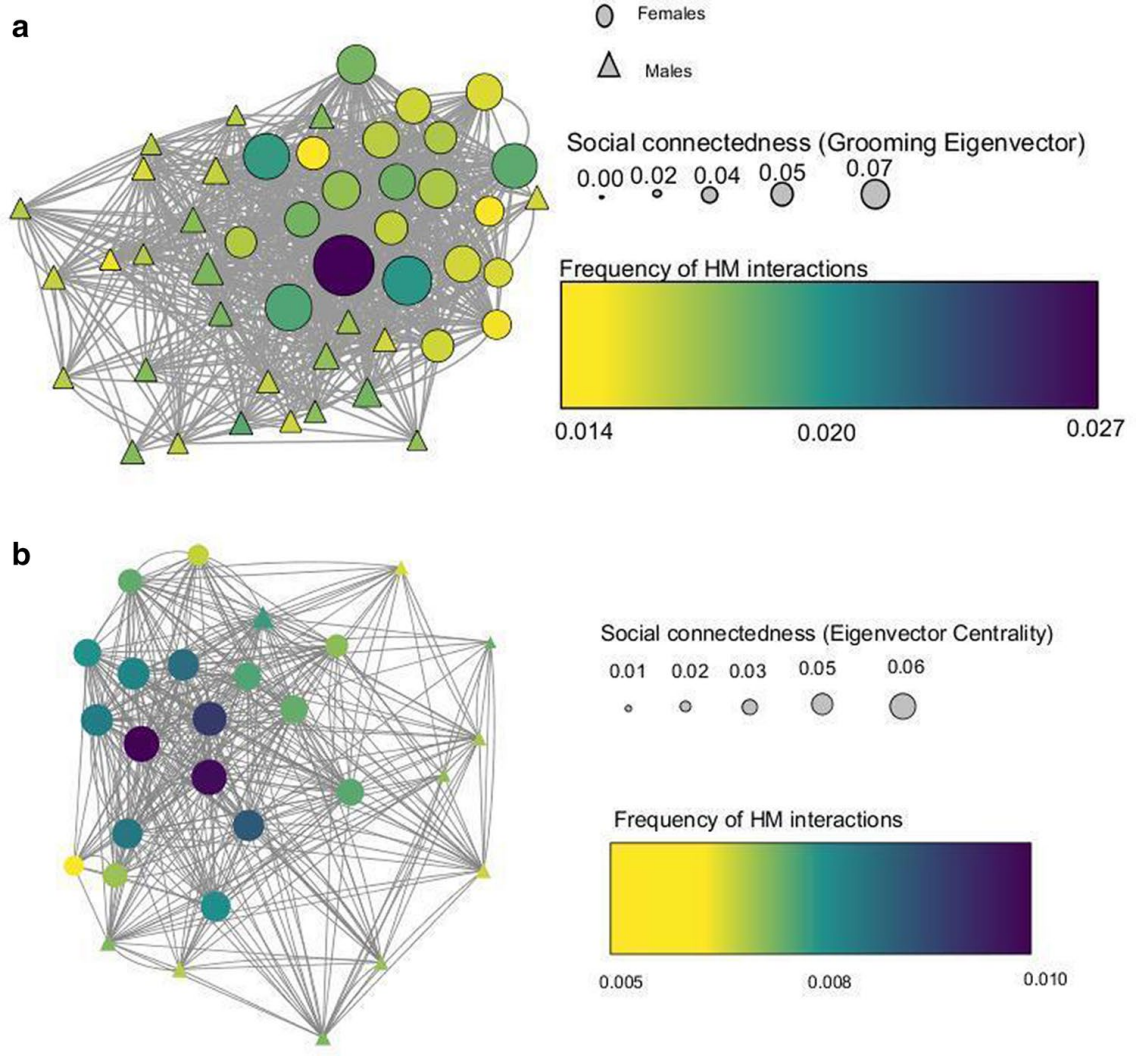
Grooming social networks of each of two bonnet macaque groups showing the effect of eigenvector centrality on the frequency of human–macaque interactions. Nodes indicate individual macaques, and are sized by grooming eigenvector centrality (larger nodes indicate higher values). Node color indicates frequencies of human–macaque interactions (darker colors indicate higher frequencies). Figure was created using Cytoscape (Version 3.7.2: https://cytoscape.org/).

The best-fit model showed significant effects of sex and spatial position, and a significant interaction between social network centrality and species, on the frequency of human–macaque interactions (Table 1; Figs. 2, 3). Specifically, males initiated more interactions than females, and spatially peripheral individuals more so than spatially central individuals (Fig. 2). The effect of social network centrality was contingent on species. Bonnet macaques showed a positive (rather than a negative, as we’d expected) relationship: central or well-connected individuals in the grooming network were engaged in more frequent interactions with humans than socially peripheral or less connected individuals (Fig. 3). On the other hand, social network centrality had no impact on frequency of interactions among either rhesus macaques or long-tailed macaques. Dominance rank showed no effect on frequency of interactions.

**Table 1:** Best-fit GLMM examining the impact of macaque attributes on the frequency of human–macaque interactions (FI).

Model equation: glmer.nb(FI ~ Sp + S + RI + SP + SC + Sp:SC + (1|G) + offset(log(OT)))				
Predictor	*B*	Std Er	z	p
(Intercept)	− 5.39	0.48	− 11.20	< 0.01**
Species (Sp) (long-tailed vs bonnet)	3.50	0.50	6.94	< 0.01**
Species (Sp) (rhesus vs bonnet)	2.15	0.50	4.26	< 0.01**
Species (Sp) (long-tailed vs rhesus)	1.35	0.39	3.43	< 0.01**
Sex (males vs females)	0.23	0.08	2.99	< 0.01**
Dominance Rank Index (RI)	− 0.07	0.11	− 0.62	0.54
Spatial Position (SP)	0.32	0.15	2.17	0.03*
Species : Social Connectedness (Sp:SC) (bonnet SC)	14.26	4.92	2.90	< 0.01**
Species : Social Connectedness (Sp:SC) (rhesus SC)	2.68	1.63	1.64	0.10
Species : Social Connectedness (Sp:SC) (long-tailed SC)	− 0.79	1.06	− 0.75	0.45

Group (G) was a random effect, and observation time (OT) was an offset variable.

**p < 0.01; *p < 0.05.

### Diversity of HM interactions

**Figure 4:**
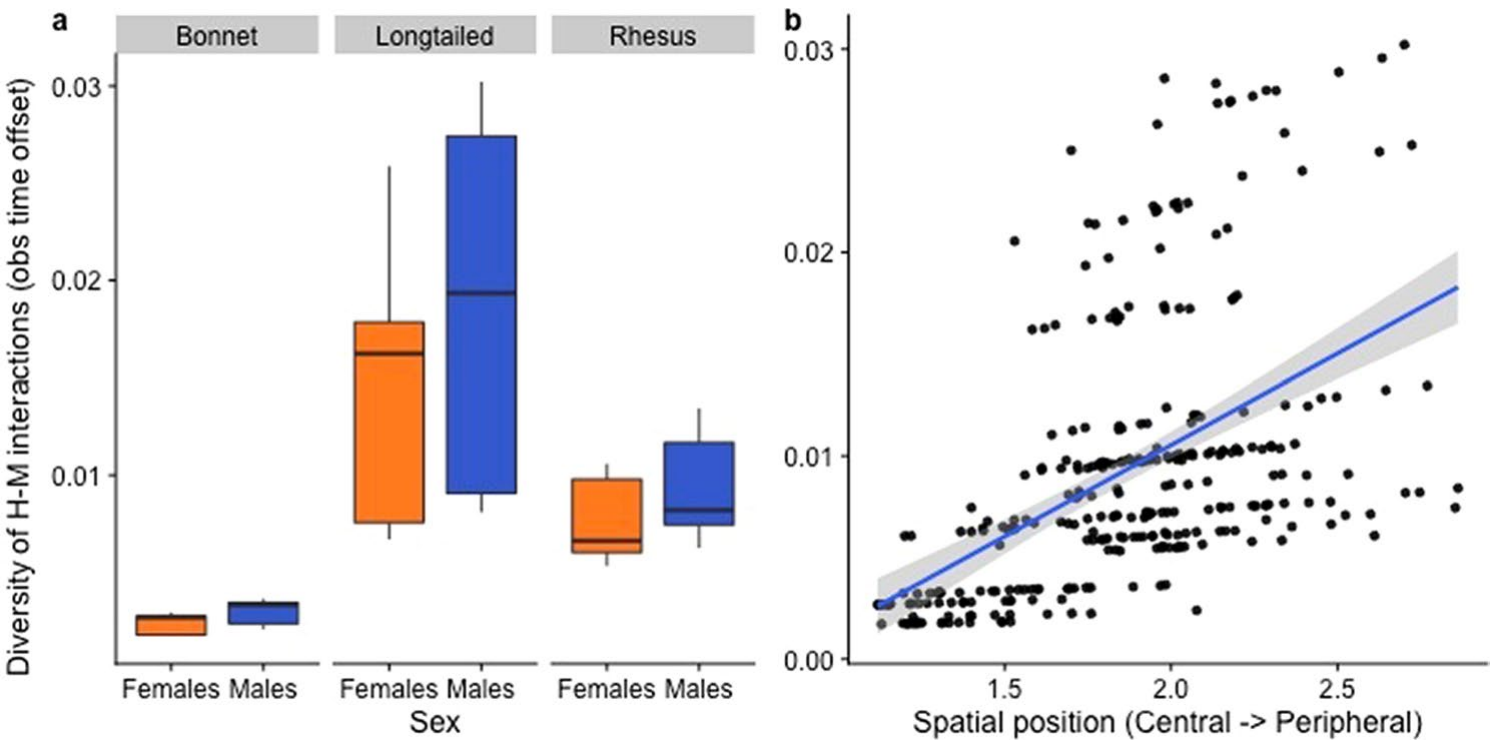
Effect of sex and species on the diversity of human–macaque interactions. As with Fig. 2, the second grouping in the scatterplot represents individuals from the long-tailed macaque groups from Batu Caves^43^. Figure was created using R (Version 3.6.1: https://www.r-project.org/).

The best-fit model revealed a significant effect of sex on diversity; males showed significantly more diverse behaviors towards humans than females (Table 2; Fig. 4). On the other hand, dominance rank, spatial position within the group, and social network centrality had no effect on diversity. We also detected significant inter-species differences in diversity: long-tailed macaques showed the most diverse behaviors, followed by rhesus macaques, and finally bonnet macaques.

**Table 2:** Best-fit GLMM examining the impact of macaque attributes on the diversity (D) of human–macaque interactions.

Model equation: glmer(D ~ Sp + S + RI + SP + SC + (1|G) + offset(log(OT)), family = "poisson")				
Predictor	*B*	Std Er	t	p
(Intercept)	− 6.34	0.32	− 20.10	< 0.01**
Species (Sp) (long-tailed vs bonnet)	1.652	0.32	5.14	< 0.01**
Species (Sp) (rhesus vs bonnet)	1.04	0.32	3.23	< 0.01**
Species (Sp) (long-tailed vs rhesus)	0.61	0.25	2.47	0.01*
Sex (male vs female)	0.18	0.05	3.32	< 0.01**
Dominance Rank Index (RI)	− 0.02	0.09	− 0.21	0.83
Spatial Position (SP)	0.16	0.11	1.51	0.13
Social Connectedness (SC)	0.36	0.59	0.60	0.55

Group (G) was a random effect, and observation time (OT) was an offset variable.

**p < 0.01.

### Complexity of HM interactions

**Figure 5:**
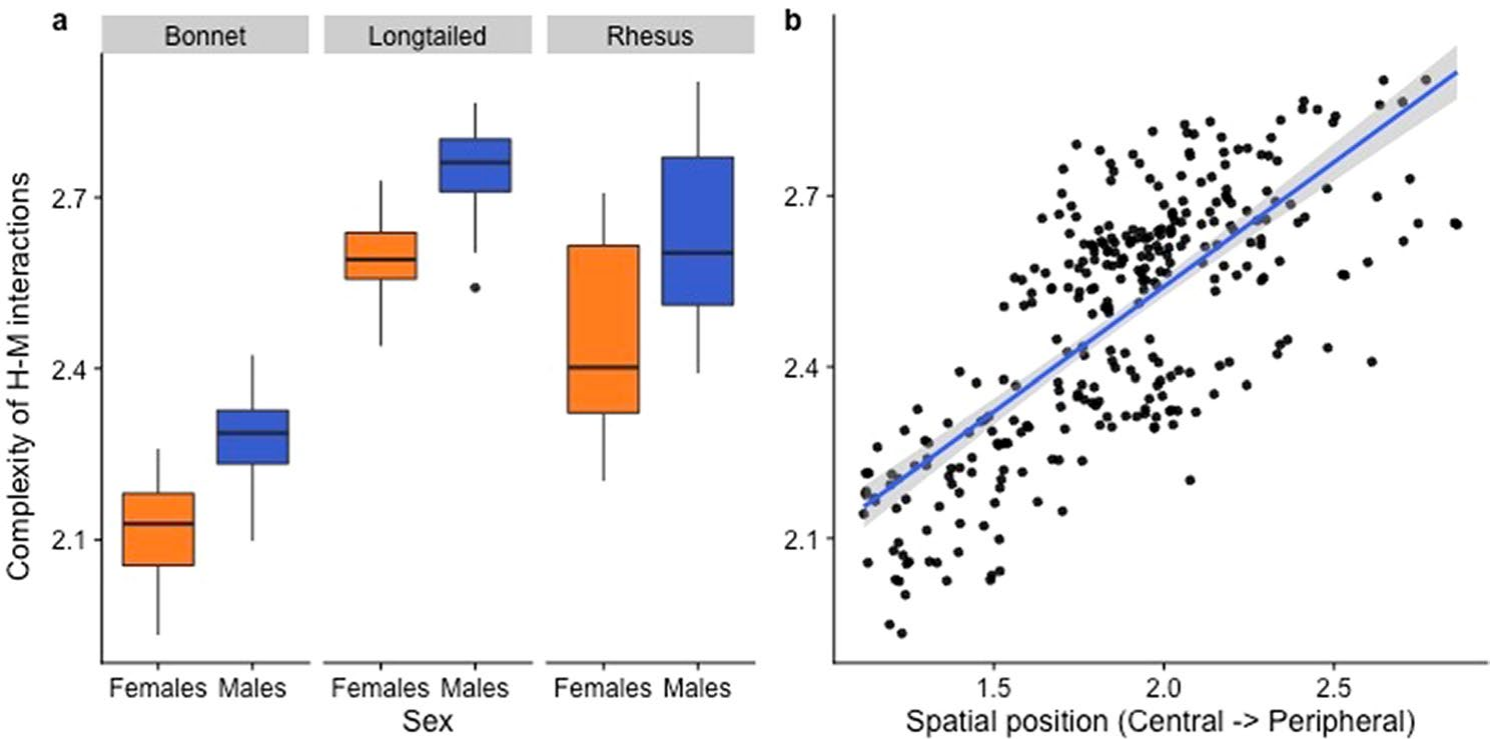
Effect of sex, species and spatial position on the complexity of human–macaque interactions. Figure was created using R (Version 3.6.1: https://www.r-project.org/).

The best-fit model showed that both sex and spatial position significantly predicted the complexity of HM interactions (Table 3; Fig. 5). Males, compared to females, were involved in more complex behaviors towards humans. Moreover, individuals in the periphery of their group engaged in more complex behaviors towards humans than those in the center. Dominance rank had no effect on complexity, nor was there any significant interaction between species and social network centrality on species (Table 3).

**Table 3:** Best-fit GLMM examining the impact of macaque attributes on the complexity (C) of human–macaque interactions.

Model equation: lmer(C ~ Sp + S + RI + SP + SC + SC:Sp + (1|G))				
Predictor	*B*	Std Er	t	p
(Intercept)	1.66	0.22	7.72	< 0.01**
Species (Sp) (long-tailed vs bonnet)	0.46	0.17	2.77	0.01*
Species (Sp) (rhesus vs bonnet)	0.23	0.17	1.34	0.20
Species (Sp) (long-tailed vs rhesus)	0.24	0.12	2.03	0.07(*)
Sex (males vs females)	0.13	0.06	2.42	0.02*
Dominance Rank Index (RI)	0.08	0.08	1.01	0.32
Spatial Position (SP)	0.23	0.10	2.30	0.02*
Species : Social Connectedness (Sp:SC) (bonnet SC)	2.62	3.09	0.85	0.40
Species : Social Connectedness (Sp:SC) (rhesus SC)	0.85	1.24	0.68	0.50
Species : Social Connectedness (Sp:SC) (long-tailed SC)	-0.88	0.82	− 1.08	0.28

Group (G) was a random effect.

**p < 0.01; *p < 0.05; (*)0.05 < p < 0.1.

### Frequency of macaque-to-human aggression

**Figure 6:**
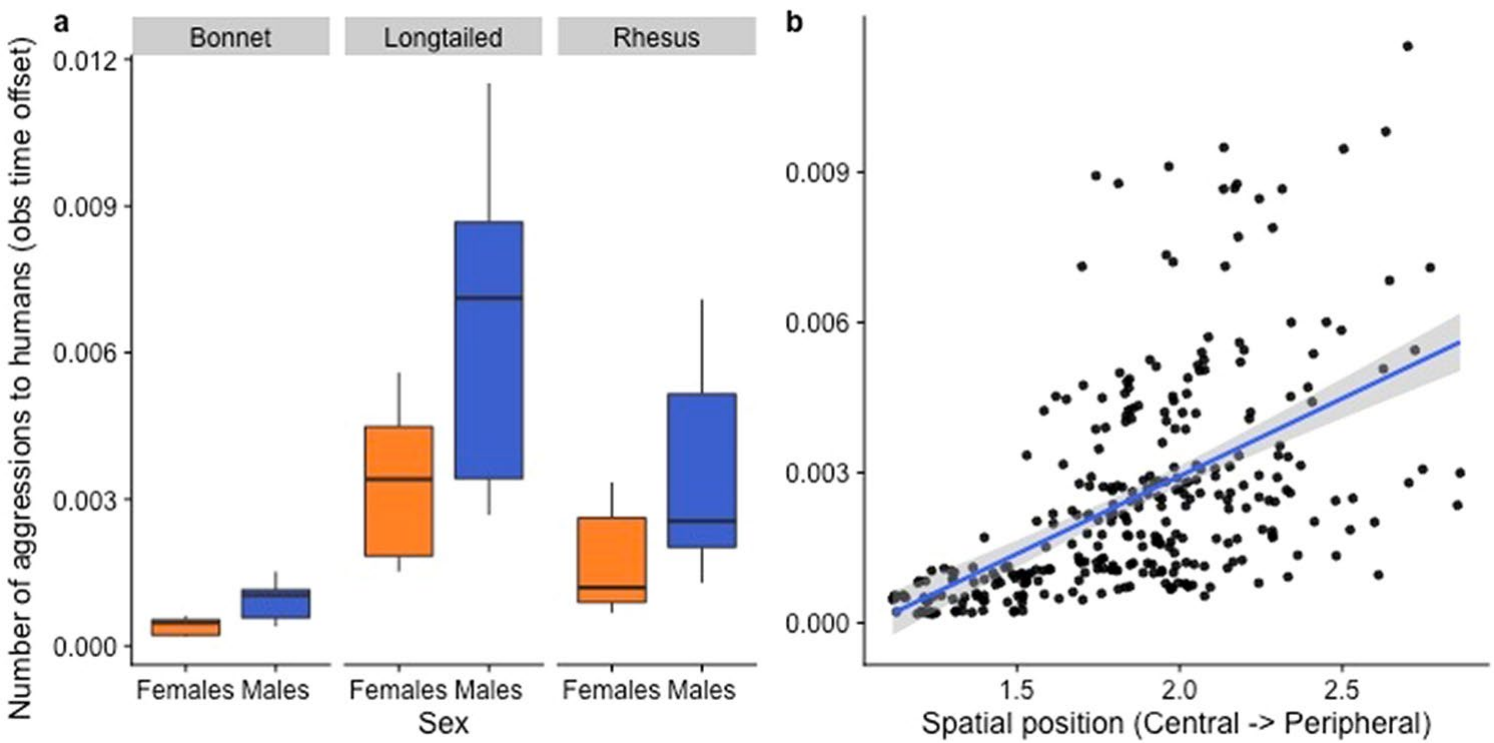
Effect of sex, species and spatial position on the frequency of macaque-to-human aggression. As with Fig. 2, the second grouping in the scatterplot represents individuals from the long-tailed macaque groups from Batu Caves^42^. Figure was created using R (Version 3.6.1: https://www.r-project.org/).

As with complexity, both sex and spatial position significantly influenced the frequency of macaque-to-human aggression (Table 4; Fig. 6). Males were more aggressive than females towards humans. More spatially peripheral individuals showed significantly more aggression towards humans than more spatially central individuals (Table 4). Neither dominance rank nor social network centrality significantly impacted macaque-to-human aggression. As with overall frequency and diversity of HM interactions, there were interspecies differences in macaque-to-human aggression: long-tailed macaques showed the highest rates, followed by rhesus macaques, and finally bonnet macaques.

**Table 4:** Best-fit GLMM examining the impact of macaque attributes on the frequency of macaque-to-human aggression.

Model equation: glmer.nb(MA ~ Sp + S + RI + SP + SC + (1|G) + offset(log(OT)))				
Predictor	*B*	Std Er	z	p
(Intercept)	− 8.88	0.55	− 16.01	< 0.01**
Species (Sp) (long-tailed vs bonnet)	1.79	0.50	3.56	< 0.01**
Species (Sp) (rhesus vs bonnet)	0.93	0.51	1.83	0.07(*)
Species (Sp) (long-tailed vs rhesus)	0.86	0.37	2.35	0.02*
Sex (males vs females)	0.60	0.11	5.53	< 0.01**
Dominance Rank Index (RI)	0.27	0.19	1.43	0.15
Spatial Position (SP)	0.57	0.22	2.61	0.01*
Social Connectedness (SC)	− 0.23	1.37	− 0.16	0.87

Group (G) was a random effect, and observation time (OT) was an offset variable.

**p < 0.01; *p < 0.05; (*) 0.05 < p < 0.1.

### Proportion of macaque-initiated *HM interactions*

**Figure 7:**
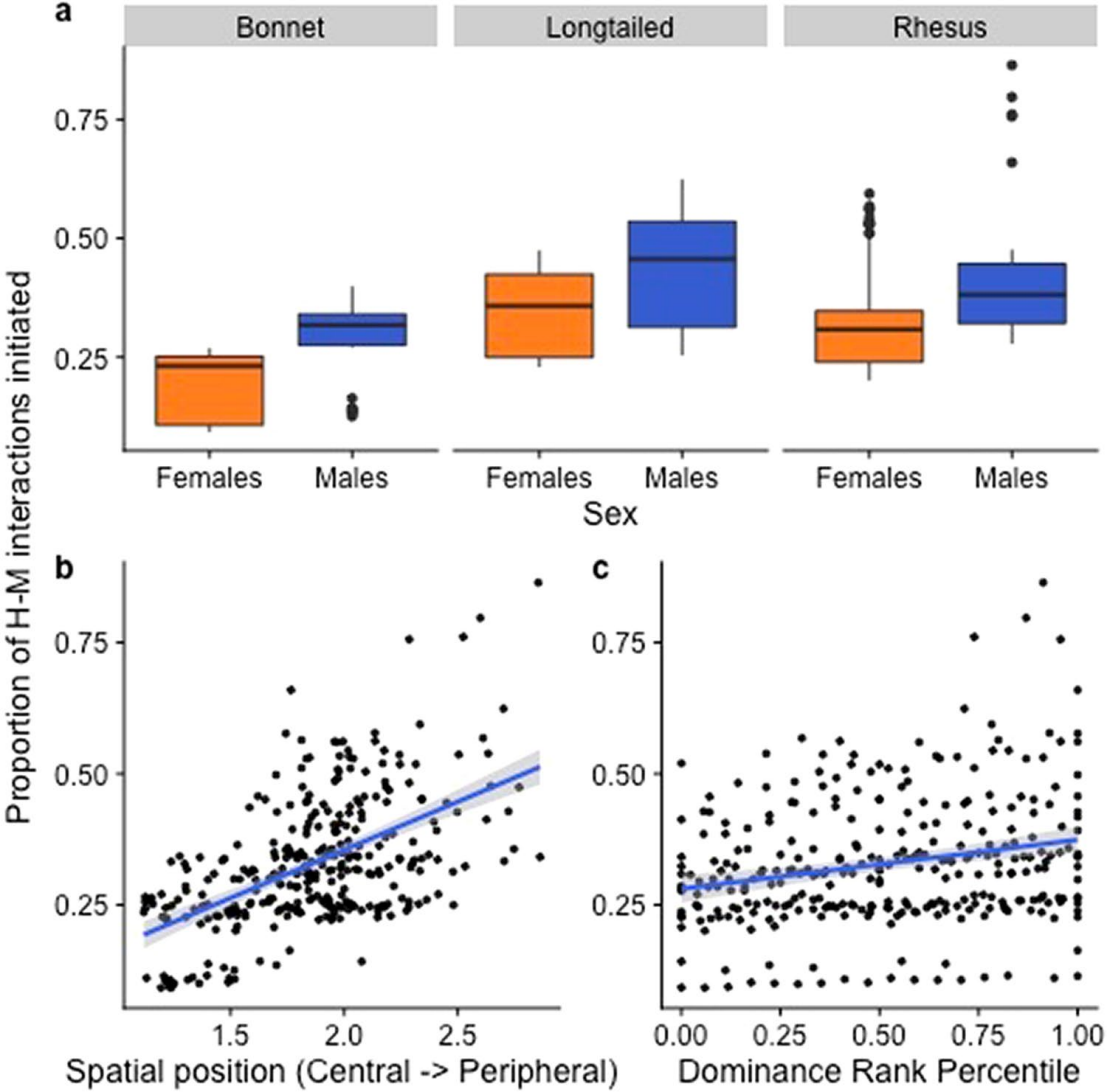
Effect of sex, species, spatial position, and dominance rank on the proportion of human–macaque interactions initiated by macaques. Figure was created using R (Version 3.6.1: https://www.r-project.org/).

The best-fit model showed significant effects of sex, spatial position, and additionally dominance rank, on the proportion of macaque-initiations (Table 5; Fig. 7). Specifically, males initiated more interactions than females, and spatially peripheral individuals more so than spatially central individuals. As predicted, dominance rank showed a positive relationship with macaque initiations, with high-ranking individuals initiating more interactions with humans than low-ranking individuals. Social network centrality had no impact on macaque initiations.

**Table 5:** Best-fit GLMM examining the impact of macaque attributes on the proportion of macaque-initiated human–macaque interactions (PMI).

Model equation: glmer.nb(PMI ~ Sp + S + RI + SP + SC + SC:Sp + (1|G) + offset(log(FI)))				
Predictor	*B*	Std Er	Z	p
(Intercept)	− 2.30	0.33	− 6.98	< 0.01**
Species (Sp) (long-tailed vs bonnet)	0.51	0.33	1.55	0.12
Species (Sp) (rhesus vs bonnet)	0.50	0.33	1.52	0.13
Species (Sp) (long-tailed vs rhesus)	0.01	0.24	0.04	0.97
Sex (males vs females)	0.22	0.06	3.64	< 0.01**
Dominance Rank Index (RI)	0.22	0.10	2.27	0.02*
Spatial Position (SP)	0.27	0.12	2.30	0.02*
Social Connectedness (SC)	0.32	0.70	0.46	0.64

Group (G) was a random effect, and the frequency of human–macaque interactions (FI) was an offset variable.

**p < 0.01; *p < 0.05.

In Table 6, we summarize the main results from each best-fit model.

**Table 6:** Summary of results from the best-fit GLMMs for each aspect of human–macaque interactions.

Outcome (distribution)	Sex	Dominance RANK	Spatial centrality	Social network centrality	Species
Frequency (negative binomial)	**Males > Females**	No significant effect	**Negative correlation**	**Positive correlation in bonnet**	**Long-tailed > rhesus > bonnet**
Diversity (Poisson)	**Males > Females**	No significant effect	No significant effect	No significant effect	**Long-tailed > rhesus > bonnet**
Complexity (Gaussian)	**Males > Females**	No significant effect	**Negative correlation**	No significant effect	**Long-tailed > rhesus and bonnet**
Aggression (negative binomial)	**Males > Females**	No significant effect	**Negative correlation**	No significant effect	**Long-tailed > rhesus > bonnet**
% Initiations (negative binomial)	**Males > Females**	**Positive correlation**	**Negative correlation**	No significant effect	No significant differences

Entries in bold font indicate significant effects (p < 0.05).

## Discussion

We adopted a comparative approach to reveal that key life-history (sex) and social traits (dominance rank, spatial position, and social network connectedness) of individuals impacted aspects of interactions between wildlife and humans in (peri)urban environments. To our knowledge, this is the first empirical assessment of individual behavior in human–wildlife interactions across locations and species. Across 10 groups of three macaque species, we found that males (compared to females) and spatially peripheral (compared to more central) individuals showed the highest indicators of tendencies to interact with humans. Across groups and species, high-ranking macaques were more likely to initiate interactions with humans. In bonnet macaques, but not in rhesus and long-tailed macaques, we found that individuals that were well-connected or central in their social grooming network showed a greater frequency of interactions with humans than less well-connected individuals. For wild primates living in anthropogenic environments, it is generally agreed that the majority of interactions with humans, if not directly involving human provisioning or primate foraging on human foods, may occur around the *context* of human defense of food resources and/or primates’ intentions to procure anthropogenic foods^25,34,78^. In this light, we discuss these findings (summarized in Table 6) in the order of the extent of impact of each life-history/social variable on aspects of human–macaque interactions, and their implications for understanding human–wildlife interfaces from evolutionary and conservation perspectives.We found that male macaques were more likely to interact with humans than females, a finding that is consistent with previous studies on other primate populations/species^28,29^ and other wildlife species^10,13^ in anthropogenic environments. Across macaque species, males showed more frequent interactions and diverse behaviors towards humans, engaged in more complex interactions (i.e. exchanged more behaviors on average) with humans, and showed both greater aggression and tendencies to initiate interactions towards humans, than females. These findings lend support to our prediction that male exploratory behavior and life-history strategies may entail greater risk-taking. One of the primary motivations behind wildlife risk-taking in anthropogenic environments is increased access to anthropogenic foods^18,34^. Although wildlife in (peri)urban landscapes may rely on environmental sources of anthropogenic food, such sources may be limited in their availability or monopolizability, particularly for group-living animals such as primates^34^. It is therefore conceivable that for males, risk-taking strategies, particularly engaging in prolonged interactions, using aggression, and initiating interactions with humans, culminates in gaining benefits related to access/monopoly of high-energy anthropogenic foods (as has already been established in our study populations:^34^). For males, the benefits of procuring such foods, manifested through the maintenance of physical features that are key to their competitive ability and reproductive success, may out-weigh costs such as receiving aggression from humans and the energy spent in gaining access to these foods. In comparison to females, where competitive ability and its associated fitness benefits are largely dependent on kinship^79^, male competitive ability and fitness are more dependent on physical features, such as, in the case of macaques, their body size/weight^80^. Thus, increased access to anthropogenic food may directly lead to increased reproductive success in males, an effect that might be especially pronounced in wildlife species like macaques where male–male competition and male reproductive skew are both high^80^.

Compared to males, females may rely more on environmental sources of anthropogenic and natural food, or may be the favored targets of human-to-macaque provisioning either because of human attraction to infants or lower perceived risk of aggression from females^28,34^. Any of these scenarios would involve lower risk-taking and energetic costs and may hence explain the lower frequency and diversity of interactions with humans, complexity of human–macaque interactions, aggression towards humans, and initiations of interactions with humans, among females. To better understand female strategies in anthropogenic environments in response to their energetic demands and life-history, comparing their interactions with humans during periods of relatively higher (pregnancy, lactation) versus lower (post-weaning) energetic costs would be a logical next step.

Among social factors, we found that individuals’ spatial position in the group had the strongest and most consistent impact on aspects of human–macaque interactions. Across groups and species, individuals that spent more time in the periphery of their group seemed to engage in more frequent and complex interactions, showed greater aggression towards humans, and initiated more interactions with humans, than more central individuals. Moreover, these effects of spatial position were independent of the effects of individuals’ dominance rank and social network centrality, neither of which had as strong or as consistent an impact (exceptional results are discussed below). In group-living animals that show non-random social structure, individuals’ spatial position within the group may be correlated to their dominance status (high-ranking individuals are often at the core of the group:^81,82^) and/or may influence their connectedness or interactions within a social network (socially well-connected individuals are also more likely to be in the core of the group:^55,83,84^). Nonetheless, spatial position within the group independently evaluated from social interactions has not been accounted for in previous studies evaluating the effects of dominance rank (Barbary macaques:^22^) or social network centrality (moor macaques:^11^) on human–primate interactions. Here we evaluated group spatial position for socially structured groups/species of wildlife, and, ruling out strong collinearity with rank and social network centrality (see “Methods”), assessed the relative effects of these three factors on human–wildlife interactions. Our finding that spatial position had a stronger, more consistent impact on human–macaque interactions than rank and/or social network connectedness is of broad relevance. Since spatial position is a fundamental aspect of all group-living animals, we speculate that our results on macaques may be generalizable to other group-living wildlife. In other words, position within a group may fundamentally underlie inter-individual variation in human–wildlife interactions across a wide range of group-living wildlife taxa in anthropogenically impacted environments, irrespective of their social complexity (e.g., white-tailed deer, *Odocoileus virginianus*^85^, elephants^13^, primates^11^).

There may be multiple, inter-dependent explanations for why peripheral macaques interacted more with humans. Simply, individuals at the edge of the group may have a greater exposure to humans than those at the center (herd effect^86^), which may explain their greater overall frequencies of interactions with humans. Alternatively, macaques of specific sociodemographic characteristics may be either forced to stay in the periphery of their group or make active decisions to stay in the periphery^81,87^. In large groups of animals where spatial position may depend on social status, low-ranking individuals with lower priority of access to natural food sources such as fruiting trees that are also not widespread or abundant in (peri)urban environments may be forced to stay in the periphery of the group and to initiate more interactions with humans to procure food. Individuals who are less attractive social partners, i.e. spend less time engaging in social interactions, may also be forced to stay in the periphery, which may increase the time available to engage in more complex interactions with humans. This argument is supported by our detection of a weak but positive correlation between spatial position centrality and grooming social network centrality (Pearson’s R: *n* = 319,* r* = 0.11,* p* = 0.05), and by our previous work that revealed an inverse effect of anthropogenic factors leading to a decrease in individuals’ time spent grooming^42–44^.

Macaques may actively choose to stay in the periphery in order to specifically interact with humans. For instance, high dominance rank and/or social connectedness may afford individuals spatial benefits in terms of being able to choose their position, with low-ranking individuals making the best out of the situation (as has been shown in wild Capuchin monkeys, *Cebus capucinus*:^81,87^). Per this explanation, peripheral macaques may also be high-ranking individuals, and/or those choosing to compromise on time spent engaging in social interactions, in order to preferentially engage in interacting with humans. Indeed, a positive correlation between dominance rank and increased access to anthropogenic food that establishes the value of anthropogenic foods supports this ‘active choice’ argument. Finally, a sub-set of peripheral individuals may constitute younger, more exploratory males that are actively seeking dispersal opportunities^88,89^ and routinely showing increases in hormonal activity (e.g., testosterone levels^90^), both of which may increase rates of aggression towards humans and/or the initiation of interactions with humans. In summary, determining whether or how animals’ decision-making regarding their spatial position impacts their behavior in these urban environments requires future analyses that (i) determine correlations between spatial position and macaques’ sociodemographic characteristics (rank, social connectedness, and sex), and (ii) examines their interactive effects on human–macaque interactions.

Compared to spatial position, the impact of dominance rank and social network centrality on aspects of human–macaque interactions was less consistent. Across species, we found a positive relationship between dominance rank and macaque initiations of human–macaque interactions: high-ranking individuals initiated more interactions with humans than low-ranking individuals. This was consistent with our prediction, but somewhat in contrast to previous findings on tourist-provisioned bonnet macaques that showed no correlation^40^, and Barbary macaques that showed a negative (rather than a positive) correlation between rank and rates of interactions with humans^22^. Our study focused on macaques in (peri)urban environments for whom being high-ranking also confers the pay-off of greater access to, and monopoly over, anthropogenic food^34^. This may compensate for the energetic costs involved in taking risks to engage with humans to procure such foods. In contrast, the afore-mentioned studies on bonnet macaques and Barbary macaques focused on wild [rather than (peri)urban] populations that were occasionally provisioned by tourists. Therefore, these animals may covet, or rely more on, natural foods than our (peri)urban macaques, making priority of access to tourist-provisioned food somewhat less relevant.

We found a conditional effect of social network connectedness on human–macaque interactions. Among bonnet macaques, but not in rhesus or long-tailed macaques, we found that centrality in the social grooming network was positively correlated to the overall frequency of interactions with humans. This finding was contrary to our original prediction of a negative relationship, based on our previous findings that anthropogenic factors led to a decrease (rather than an increase) in grooming duration and (to a lesser extent) partner diversity in these bonnet macaques^44^. The reason for this could be that in those previous studies, we had evaluated grooming effort by season, whereas here we evaluated individuals’ grooming eigenvector centrality (and not just grooming duration or diversity) across the entire study period. Eigenvector is a complex, network measure of the strength and diversity of an individuals’ direct *and* secondary connections, or long-term ‘social capital’^54,91^. Previous studies on primates and other group-living animals have shown that increased eigenvector centrality may be associated with stronger social bonds, greater access to resources, and increased social support during intra-group conflict (reviewed in^92^). Given its clear significance, it is likely that inter-individual variation in macaques’ eigenvector centrality (i) may be conserved in spite of time-budgets leading to a systematic, group-wide reduction in grooming effort, and moreover (ii) reduce/negate the potential costs of risky behaviors such as engagement with humans. Such effects, we speculate, may be especially pronounced among primate groups/species which (i) display more tolerant social systems that are characterized by strong, diverse affiliative relationships (bonnet macaques > rhesus or long-tailed macaques:^58^), and/or (ii) have historically experienced relatively lower levels of anthropogenic impact (bonnet macaques < rhesus or long-tailed macaques:^64^).

At the species level, we found that long-tailed macaques consistently showed the most frequent indicators of interactions with humans, followed by rhesus macaques, and finally bonnet macaques. In general, comparative assessments of human–wildlife interfaces would require a careful consideration of those wildlife and human components that are similar, versus those that are different^3,5^. Here we focused on three macaque species that bore systematic similarities and differences in their evolutionary history and ecological flexibility (^58^, see “Introduction”). One explanation for such differences might be species-typical variation based on their phylogenetic distances: long-tailed macaques and rhesus macaques are more closely related to each other, are more geographically and ecologically widespread, and have experienced a longer history of exposure to anthropogenic environments, than bonnet macaques (^58^, see “Introduction”). A more robust test of this explanation awaits the implementation of phylogenetic comparative methods. Implementing phylogenetic approaches were beyond the scope of this study, since our data were limited to three species, and given well-established empirical evidence that such methods are highly susceptible to low sample sizes of the number of species^93–95^. Our findings should lead naturally to future assessments if or once comparable behavioral data on other primates and other wildlife living in human-impacted environments is collected or assembled.

We found that cross-species variation failed to reach significance for macaque-initiations of human–macaque interactions. This finding suggests that any explanation of species-typical or phylogenetic differences driving variation in human–macaque interactions is incomplete without a consideration of cross-site differences in current environmental conditions. In other words, a more complete picture will emerge when we evaluate and compare intraspecific variation across groups, and the role of anthropogenic environmental factors, on these human–wildlife interactions. Although we studied (peri)urban primate populations, they nonetheless experienced varying degrees of anthropogenic exposure across locations [Malaysia > Northern India > Southern India: (McCowan, *Unpublished Data*)], and even across groups within the same location (e.g. density of humans; access to anthropogenic food:^34,43^). Within Malaysia, for instance, we studied two groups that visited a Hindu temple and experienced a markedly high frequency of interactions with humans, and two others in a (peri)urban park that experienced comparatively lower frequencies^43^. Here our approach controlled for such cross-group differences within species, while quantitatively evaluating cross-species differences. A more comprehensive examination of intraspecific variation, beyond the scope of this study, would require evaluations of inter-group differences in macaque behavior while also considering aspects of the human system, specifically human demographic characteristics, attitudes, and behavior, that might influence these interactions.

Findings from this study add to a growing body of research focusing on the characteristics and behavior of individual wild animals at human–wildlife interfaces. They suggest that the life-history, group-living, and social behavior of individual wild animals, may all influence their interactions with humans. From an evolutionary perspective, our results suggest that among group-living animals in energetically challenging (peri)urban environments, individuals with (1) *more energetically costly* strategies, e.g. compromised group-living (spatial periphery) and greater exploration (males, peripheral individuals), but also with (2) the *less costly* strategies related to their life-history (males) and increased access to resources (high rank) and social capital (increased grooming connectedness within a socially tolerant group), are the most prone to risk-taking by interacting with humans. From a conservation perspective, our findings inform human–wildlife conflict and disease intervention strategies to focus on specific classes of ‘target’ individuals that may be (1) more prone to conflict-defining interacting with humans (e.g., males, peripheral animals), and consequently (2) more prone to acquiring and transmitting zoonotic agents from humans (i.e. ‘superspreader’ primates: e.g. peripheral or migrating macaques at the human–wildlife interface^16^; high-ranking or socially central individuals within wildlife social groups^96^). Such targeted efforts may reduce both the monetary and health-related costs of conflict to both wildlife and humans.

## Supplementary Information

Supplementary Information.

## Data Availability

The data used in this study is available with the first/corresponding authors and the last/senior author and will be provided upon request.
