# Supplementary material for: Impact of individual demographic and social factors on human–wildlife interactions: a comparative study of three macaque species
Source: Sci Rep. 2020 Dec 15;10:21991. doi: 10.1038/s41598-020-78881-3 (PMC7738552; doi:10.1038/s41598-020-78881-3)
Supplement: Supplementary file 1 — Supplementary Information. [file 41598_2020_78881_MOESM1_ESM.docx]

**Impact of Individual Demographic and Social Factors on Human-Wildlife Interactions: A Comparative Study of Three Macaque Species**

Krishna N. Balasubramaniam*^1^, Pascal R. Marty*^1,2^, Shelby Samartino^1,3^, Alvaro Sobrino^1,2^, Taniya Gill^1,4^, Mohammed Ismail^1,5^, Rajarshi Saha^1,6^, Brianne A. Beisner^1,7^, Stefano S. K. Kaburu^1,8^, Eliza Bliss-Moreau^7,9^, Malgorzata E. Arlet^6^, Nadine Ruppert^2^, Ahmad Ismail^10^, Sahrul Anuar Mohd Shah^2^, Lalith Mohan^11^, Sandeep Rattan^11^, Ullasa Kodandaramaiah^12^, & Brenda McCowan^1,7^

^1^Department of Population Health & Reproduction, School of Veterinary Medicine (SVM), University of California at Davis, Davis CA 95616, USA

^2^School of Biological Sciences, Universiti Sains Malaysia, 11800 Pulau Pinang, Malaysia

^3^Department of Anthropology, University of Texas at San Antonio, San Antonio TX 78249, USA

^4^Department of Anthropology, University of Delhi, Delhi 110007, India

^5^Primate Conservation Group, Oxford Brookes University, Oxford OX3 0BP, UK

^6^Adam Mickiewicz University, Institute of Human Biology and Evolution, Faculty of Biology, Poznań 61614, Poland

^7^California National Primate Research Center, University of California, Davis CA 95616, USA

^8^Department of Biomedical Science and Physiology, Faculty of Science and Engineering, University of Wolverhampton, Wolverhampton WV1 1LY, UK

^9^Department of Psychology and the California National Primate Research Center, University of California, Davis CA 95616, USA

^10^Department of Biology, Faculty of Science, Universiti Putra Malaysia, 43400 UPM Selangor, Malaysia

^11^Himachal Pradesh Forest Department, Shimla, Himachal Pradesh 171002, India

^12^IISER-TVM Centre for Research and Education in Ecology and Evolution (ICREEE), School of Biology, Indian Institute of Science Education and Research Thiruvananthapuram, Maruthamala P.O., Vithura, Thiruvananthapuram, India. 695551

*Equal contribution by co-first authors of the manuscript

Contact information of corresponding author:

Dr. Krishna N. Balasubramaniam

[krishnanatarajan@ucdavis.edu](mailto:krishnanatarajan@ucdavis.edu)

**Supplementary Table 1:** Demography of all study groups with number of males and females and the total observation time.

| Species | Group | # of Males | # of Females | Observation time (h) |
| --- | --- | --- | --- | --- |
| *Macaca mulatta* | MM1 | 6 | 17 | 275.3 |
| (Shimla, India) | MM2 | 7 | 17 | 347.2 |
|  | MM3 | 12 | 43 | 487.2 |
|  | MM4 | 12 | 25 | 662.5 |
| *Macaca fascicularis* | MF1 | 9 | 22 | 449.1 |
| (Kuala Lumpur, Malaysia) | MF2 | 6 | 12 | 252.8 |
|  | MF3 | 6 | 18 | 293.0 |
|  | MF4 | 14 | 19 | 230.4 |
| *Macaca radiata*  (Thenmala, India) | MR1 | 24 | 22 | 553.2 |
|  | MR2 | 10 | 18 | 319.8 |
